# Genome-wide survey of soybean papain-like cysteine proteases and their expression analysis in root nodule symbiosis

**DOI:** 10.1186/s12870-020-02725-5

**Published:** 2020-11-12

**Authors:** Songli Yuan, Danxia Ke, Rong Li, Xiangyong Li, Lei Wang, Haifeng Chen, Chanjuan Zhang, Yi Huang, Limiao Chen, Qingnan Hao, Hongli Yang, Dong Cao, Shuilian Chen, Wei Guo, Zhihui Shan, Zhonglu Yang, Xiaojuan Zhang, Dezhen Qiu, Yuefeng Guan, Xinan Zhou

**Affiliations:** 1 Key Laboratory of Biology and Genetic Improvement of Oil Crops, Ministry of Agriculture and Rural Affairs, Oil Crops Research Institute of Chinese Academy of Agricultural Sciences, Wuhan, China; 2 College of Resources and Environment, Fujian Agriculture and Forestry University, Fuzhou, 350002 Fujian China; 3 College of Life Sciences & Institute for Conservation and Utilization of Agro-bioresources in Dabie Mountains, Xinyang Normal University, Xinyang, China; 4 FAFU-UCR Joint Center for Horticultural Biology and Metabolomics, Fujian Agriculture and Forestry University, Fuzhou, 350002 China

**Keywords:** Genome-wide survey, Nodule development and senescence, Papain-like cysteine protease, Root nodule symbiosis, Soybean

## Abstract

**Background:**

Plant papain-like cysteine proteases (*PLCPs*) are a large class of proteolytic enzymes and play important roles in root nodule symbiosis (RNS), while the whole-genome studies of *PLCP* family genes in legume are quite limited, and the roles of *Glycine max PLCPs* (*GmPLCPs*) in nodulation, nodule development and senescence are not fully understood.

**Results:**

In the present study, we identified 97 *GmPLCPs* and performed a genome-wide survey to explore the expansion of soybean *PLCP* family genes and their relationships to RNS. Nineteen paralogous pairs of genomic segments, consisting of 77 *GmPLCPs*, formed by whole-genome duplication (WGD) events were identified, showing a high degree of complexity in duplication. Phylogenetic analysis among different species showed that the lineage differentiation of *GmPLCPs* occurred after family expansion, and large tandem repeat segment were specifically in soybean. The expression patterns of *GmPLCPs* in symbiosis-related tissues and nodules identified RNS-related *GmPLCPs* and provided insights into their putative symbiotic functions in soybean. The symbiotic function analyses showed that a RNS-related *GmPLCP* gene (*Glyma.04G190700*) really participate in nodulation and nodule development.

**Conclusions:**

Our findings improved our understanding of the functional diversity of legume *PLCP* family genes, and provided insights into the putative roles of the legume *PLCPs* in nodulation, nodule development and senescence.

**Supplementary Information:**

The online version contains supplementary material available at 10.1186/s12870-020-02725-5.

## Background

Plant papain-like cysteine proteases (PLCPs) are a large class of proteolytic enzymes associated with plant-pathogen/pest interactions, seed germination, development, immunity, senescence, cyclization and stress responses [1–8]. PLCPs belong to C1A CysProt (family C1, clan CA), and are a typical member of plant cysteine proteases [3, 9]. These enzymes are produced as inactive precursors with a signal peptide, an auto-inhibitory prodomain and an active protease domain [1]. Besides, some PLCPs carry a GRAN domain in their C-terminal regions [10]. *PLCP* family genes have been systematically studied in *Arabidopsis*, rubber, cassava, castor, poplar, grapevine, *Gossypium hirsutum*, *Carica papaya* and rice [1, 11–14], while the studies of *PLCP* family genes are very limited in the whole genome of legume.

*PLCP* family genes have been shown to participate in nodulation [15, 16] as well as nodule development and senescence in soybean, *Astragalus sinicus*, *Pisum sativum* and *Medicago truncatula* [17]. For example, *PsCYP1* is expressed at the onset of senescence in the indeterminate nodules, and *PsCyp15A* and *MsCYP15A* are expressed in nodules [18, 19]. *MtCP6* is induced to express during both developmental and stress-induced nodule senescence, and its early expression promoted nodule senescence [20]. *MtCP77* positively regulates nodule senescence by accelerating plant PCD and ROS accumulation [21]. *Asnodf32*, a nodule-specific cysteine protease [22], negatively regulate nodule development, bacteroid senescence and nodule lifespan in *A. sinicus* [23]. *Glycine max CYSP1*(*GmCYSP1*) may participate in nodule development and senescence [24]. However, these studies are mainly based on individual members of *PLCPs*. In soybean, dozens of *PLCPs* are associated with root nodule symbiosis (RNS) [25–27], while the role of *GmPLCP* in nodulation, nodule development and senescence is not fully understood.

In the present study, a whole-genome survey was performed to explore the special characteristics and the expansion of soybean *PLCP* family genes. The expression profiles of *GmPLCPs* in soybean root nodule symbiosis were analyzed to identify RNS-associated *PLCPs*. The symbiotic function analysis showed that a RNS-related *GmPLCP* gene (*Glyma.04G190700*) was likely to play roles in nodulation and nodule development. Our findings improve our understanding of the functional diversity of legume *PLCP* family genes, and provide insights into the putative roles of the legume *PLCPs* in nodulation, nodule development and senescence.

## Results

### Identification of *PLCP* gene family in soybean

Surveys of the soybean genomes preliminary identified 106 gene loci encoding putative PLCPs in the *Glycine max* var. Williams 82 genome (Table S1). The identified soybean PLCPs had various molecular masses ranging from 6128.82 to 57,479.13 Da, and they encoded peptides with 55 ~ 517 amino acid residues and isoelectric point (pI) of 4.3 ~ 9.32. Conserved domains in these 106 PLCPs were analyzed by NIH/NLM/NCBI CD-search tool, and Table S2 lists the detailed information. Inhibitor_I29 and peptidase_C1 motifs are commonly conserved in soybean PLCPs, and five PLCPs (Glyma.04G028300, Glyma.10G120700, Glyma.13G229100, Glyma.14G085800 and Glyma.17G239000) also had a GRAN motif. Besides, among these 106 soybean PLCPs, nine of them were considered as putative pseudogenes based on their sequence length (Table S1) and the absence of peptidase_C1 domain or the presence of large fragment deletion in the peptidase_C1 domain (Table S2), so we identified a total of 97 GmPLCPs in soybean.

### Chromosome location and duplication of soybean *PLCPs*

**Fig. 1:**
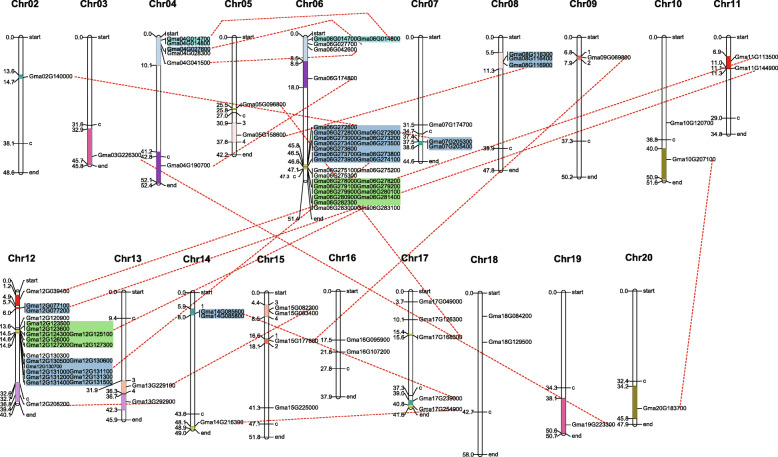
Chromosomal locations, tandem region duplication and 19 paralogous segments pairs for *GmPLCPs*. Red dotted lines indicate duplicated segments pairs. The duplicated segments generated by the most recently WGD event of soybean are indicated by different colored boxes. The chromosome number is indicated above each chromosome and *GmPLCPs* were not located in Chr01. The Map Chart and Adobe Illustrator were used to produce and modify the figure, respectively

**Fig. 2:**
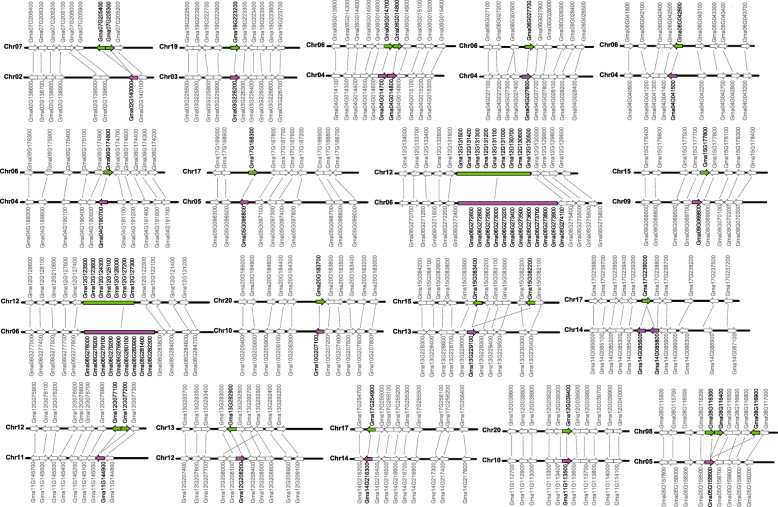
Collinearity analysis of 19 paralogous segments pairs in *GmPLCPs*. Duplicated *GmPLCP* gene pairs are indicated by Green and purple arrows or sticks. The positions of the genes in the positive (to left) and negative (to right) chain of DNA are indicated by the direction of the arrow

To survey the potential duplications of soybean *PLCPs*, firstly, candidate *GmPLCP* duplicate pairs located in a pair of paralogous blocks formed by *Glycine* WGD. As shown in Fig. 1, 19 candidate paralogous segment pairs containing 77 soybean *PLCPs* were observed on 17 soybean chromosomes. Besides, two gene clusters (*Glyma.06G275100*, *Glyma.06G275200* and *Glyma.06G275300*; *Glyma.06G283000* and *Glyma.06G283100*) were observed on chromosome 6, and *Glyma.12G130300* was localized in the big gene cluster on chromosome 12. Secondly, collinearity analysis was further carried out among candidate duplicate segment pairs, and Fig. 2 shows the flanking regions of the candidate duplicate segment pairs. Finally, 19 duplicate segment pairs formed by *Glycine* WGD events were identified (Table 1). Among them, it was worth noting that pairs 10 and 11 included large tandem repeat segments located on chromosome 6 or chromosome 12, indicating that a high degree of complexity existed in these soybean *PLCP* duplications.

**Table 1:** Divergence between soybean *PLCP* segment pairs in soybean

No.	Segment pairs		Ka	Ks	Ka/Ks	Estimated time (mya)
1	*Glyma.02G140000*	*Glyma.07G205400*	0.1883	0.3374	0.56	27.66
		*Glyma.07G205300*				
2	*Glyma.03G226300*	*Glyma.19G223300*	0.0172	0.1095	0.16	8.98
3	*Glyma.04G014700*	*Glyma.06G014700*	0.0204	0.2035	0.10	16.68
4	*Glyma.04G014800*	*Glyma.06G014800*	0.0108	0.1554	0.07	12.74
5	*Glyma.04G027600*	*Glyma.06G027700*	0.0241	0.1339	0.18	10.98
6	*Glyma.04G041500*	*Glyma.06G042600*	0.0849	0.2065	0.41	16.93
7	*Glyma.04G190700*	*Glyma.06G174800*	0.0266	0.1233	0.22	10.11
8	*Glyma.05G096800*	*Glyma.17G168300*	0.0322	0.1257	0.26	10.30
9	*Glyma.05G158600*	*Glyma.08G116300*	0.0832	0.1294	0.64	10.61
		*Glyma.08G116400*				
		*Glyma.08G116900*				
10	*Glyma.06G272600*	*Glyma.12G130500*		0.196		16.07
	*Glyma.06G272800*	*Glyma.12G130600*				
	*Glyma.06G272900*	*Glyma.12G130700*				
	*Glyma.06G273000*	*Glyma.12G131100*				
	*Glyma.06G273200*	*Glyma.12G131400*				
	*Glyma.06G273400*	*Glyma.12G131300*				
	*Glyma.06G273500*	*Glyma.12G131500*				
	*Glyma.06G273600*	*Glyma.12G131000*				
	*Glyma.06G273700*	*Glyma.12G131200*				
	*Glyma.06G273800*					
	*Glyma.06G273900*					
	*Glyma.06G274100*					
11	*Glyma.06G278000*	*Glyma.12G127200*		0.1558		12.77
	*Glyma.06G278200*	*Glyma.12G127300*				
	*Glyma.06G279100*	*Glyma.12G126000*				
	*Glyma.06G279200*	*Glyma.12G124300*				
	*Glyma.06G279900*	*Glyma.12G125100*				
	*Glyma.06G280100*	*Glyma.12G123500*				
	*Glyma.06G280900*	*Glyma.12G123600*				
	*Glyma.06G281400*					
	*Glyma.06G282300*					
12	*Glyma.09G069800*	*Glyma.15G177800*	0.0334	0.2129	0.16	17.45
13	*Glyma.10G207100*	*Glyma.20G183700*	0.0271	0.1038	0.26	8.51
14	*Glyma.11G113500*	*Glyma.12G039400*	0.0433	0.1597	0.27	13.09
15	*Glyma.11G144900*	*Glyma.12G077100*	0.0397	0.1028	0.39	8.43
		*Glyma.12G077200*				
16	*Glyma.12 g208200*	*Glyma.13 g292900*	0.1635	0.2831	0.58	23.20
17	*Glyma.13G229100*	*Glyma.15G082200*	0.093	0.1961	0.47	16.07
		*Glyma.15 g083400*	0.1398			
18	*Glyma.14G216300*	*Glyma.17G254900*	0.0315	0.1857	0.17	15.22
19	*Glyma.17G239000*	*Glyma.14G085800*	0.0255	0.1713	0.15	14.04
		*Glyma.14G085600*				

The Soybean Genome Database was used to search the synonymous mutation rate (Ks) values of these *PLCPs*. All Ks values ranged from 0.1028 to 0.3374, and the divergence times of these 19 duplicate paralogous pairs ranged from 8.43 and 27.66 Mya, with an average of 14.2 Mya (Table 1), which was consistent with WGD events (10–20 Mya). The ratio of non-synonymous mutation rate (Ka) to Ks (ω = Ka/Ks) is usually used to measure the history of selection acting on coding sequences [28]. When ω < 1, at least one gene is under purifying selection, whereas ω > 1 suggests directional selection [29, 30]. Table 1 shows that ω ranged from 0.069 to 0.643 for 17 duplicate segment pairs (except for No. 10 and No. 11), suggesting that these genes were constrained by purifying selection.

### Phylogenetic and exon-intron structure analysis of *PLCPs* in *Arabidopsis thaliana*, *M. trunctula*, *Lotus japonicus* and soybean

**Fig. 3:**
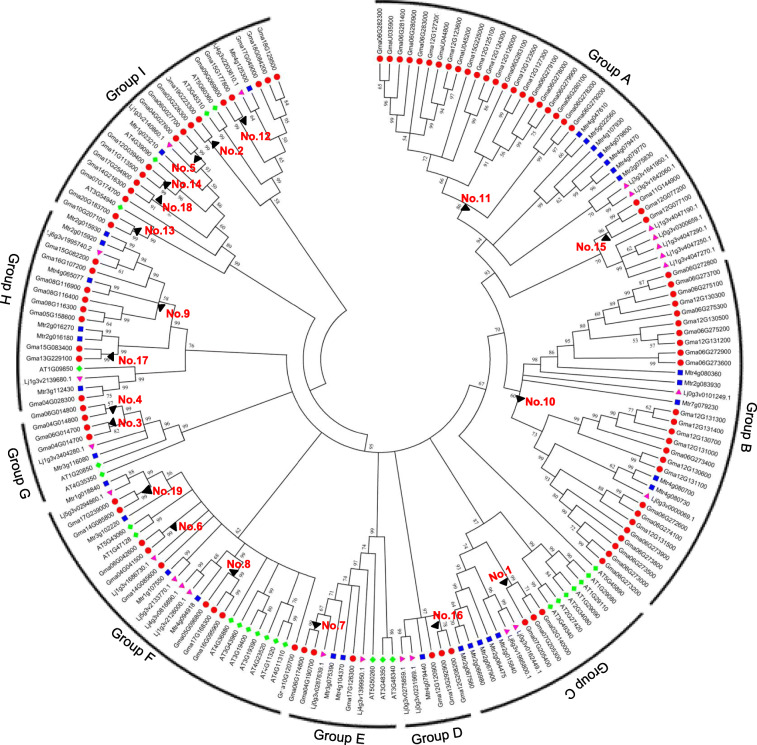
Phylogenetic relationships among the *PLCPs* from soybean, *L. japonicus*, *M. trunctula* and *A. thaliana*. The program MEGA6.0 was used to construct the phylogenetic tree, which including 97 *PLCPs* from soybean, 25 *PLCPs* from *L. japonicus*, 33 *PLCPs* from *M. trunctula* and 26 *PLCPs* from *A. thaliana*. These *PLCPs* were classified into nine groups, namely Group A to Group I. Gene duplication event marked with “ ”

To investigate the phylogenetic relationship of soybean *PLCPs* with those of other legume plants and non-legume plants, we conducted a full-length peptide sequence alignment among 97 *GmPLCPs* (Soybean), 26 *AtPLCPs* (*A. thaliana*), 33 *MtPLCPs* (*M. trunctula*), and 25 *LjPLCPs* (*L. japonicus*) using the MEGA (version 6.0) (Fig. 3). The *PLCPs* from these four different species were distributed in nine groups (Group A to Group I). Among them, soybean *PLCPs* and 19 duplicate segment pairs formed by *Glycine* WGD events were distributed in all the nine groups, indicating that the lineage differentiation of soybean *PLCPs* occurred after family expansion. The soybean *PLCP* members were not evenly distributed in these nine groups, and among them, Group A, B and Group D were formed with only legume *PLCPs* (Fig. 3), indicating that these three groups occurred before the differentiation between *A. thaliana* and legumes or were lost in *A. thaliana* or specific in legumes. Besides, *AT5G45890* was independent of each group, indicating that this gene had species-specific characteristics and new functions.

Among the *PLCPs* from the three legume plants, although tandem repeat events were found in *M. trunctula* and *L. japonicus,* large tandem repeat segment pairs only existed in soybean *PLCPs* (pairs No. 10 and 11 in group A and B), indicating that this complex replication of *PLCPs* was specific for soybean, not for legume. The number of *PLCPs* in soybean was significantly more than that in *M. trunctula* and *L. japonicus*, in addition to the above-mentioned large tandem repeat segment events, the reason could be mainly attributed to fragment repeat events in soybean, especially in groups G and I (Fig. 3). In these two groups, there were only three *MtPLCPs* and three *LjPLCPs*, while there were 20 paralogous soybean *PLCPs*. However, it remained largely unexplored why these paralogous genes were retained in soybean, which might be associated with some biological functions.

**Fig. 4:**
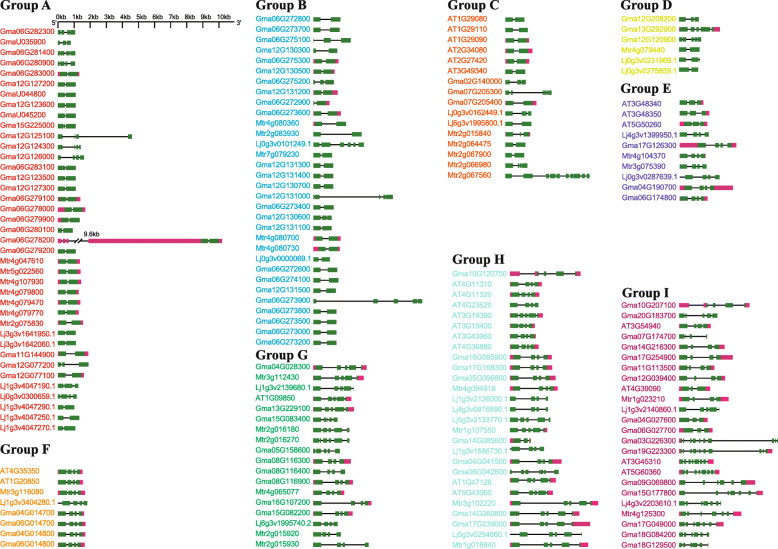
Exon/intron structures of *PLCPs* from soybean, *L. japonicus*, *M. trunctula* and *A. thaliana*. 181 *PLCPs* were classified into nine groups, namely Group A to Group I (according to the classification in Fig. 2). The pale green box represents exons, the plum red represents UTR, the black line refers introns, slashes represent over-longed introns, and the scales at the top are used to estimate the sizes of exons and introns

The cDNA sequence of each *PLCP* in above-mentioned nine groups (Fig. 3) was compared with their genomic sequences to analyze their UTR/exon/intron structures, and similar gene structures were found within each group among these *PLCPs* (Fig. 4). Most of the *PLCPs* in Group A to Group D exhibited relatively simple gene structures, among them, 70% *PLCPs* contained only one intron. While in Group E to Group I, all *PLCPs* in these five groups harbored two or more introns (Fig. 4). These group-specific gene structures were consistence with the relationships between *PLCPs* across species and further supported functional divergence among these *PLCPs*.

### Go analysis of soybean *PLCPs*

**Fig. 5:**
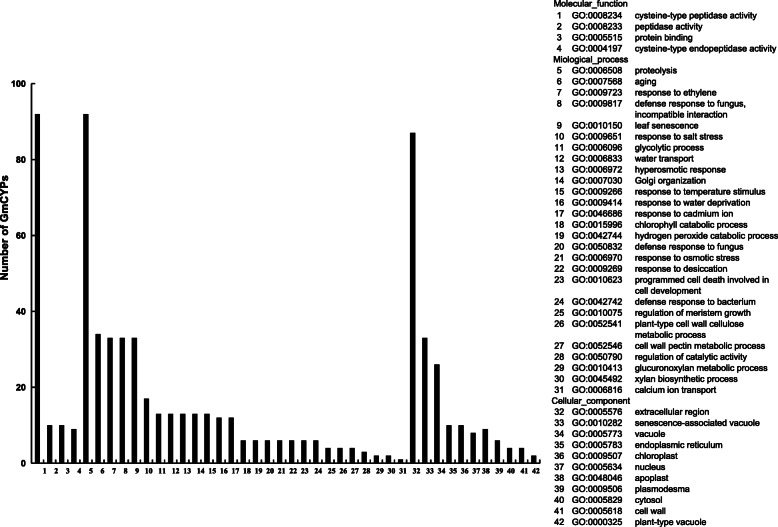
Gene Ontology - based functional annotation of *GmPLCPs*. The three GO domains - molecular function (1–4), biological process (5–31), cellular components (32–42) are shown. The numbers of genes in each term are shown in histograms

Go of the soybean *PLCPs* was investigated based on the putative assignment of 42 Go terms using the data in Soybean Genome Database. These Go function terms were divided into three categories: biological process, cellular components, and molecular function (Fig. 5), and the detailed gene ID information of them was shown in Table S3. Among them, five genes (*Glyma.06G042600*, *Glyma.06G282300*, *Glyma.12G124300*, *Glyma.12G126000* and *Glyma.12G131000*) had no predicted Go function. All of the rest had cysteine-type peptidase activity and participated in the proteolysis process, and most of the soybean *PLCPs* were localized in extracellular region (87 genes). For the duplicate segment pairs, two genes in pair 1, three genes in pair 15 and 23 genes in the two most complex pairs (pairs NO. 10 and 11) participated in the aging, response to ethylene, defense response to fungus, incompatible interaction and leaf senescence processes, and had senescence-associated vacuole location. For the four pairs (No. 1, 9, 17 and 19), one of the tandem repeat genes of them has different function from the others. Besides, for the rest 12 gene pairs, except for pair 6 (*Glyma.06G042600* had no predicted Go function), both of the two genes participated in the same biological processes.

### Expression patterns of soybean *PLCPs* in RNS

**Fig. 6:**
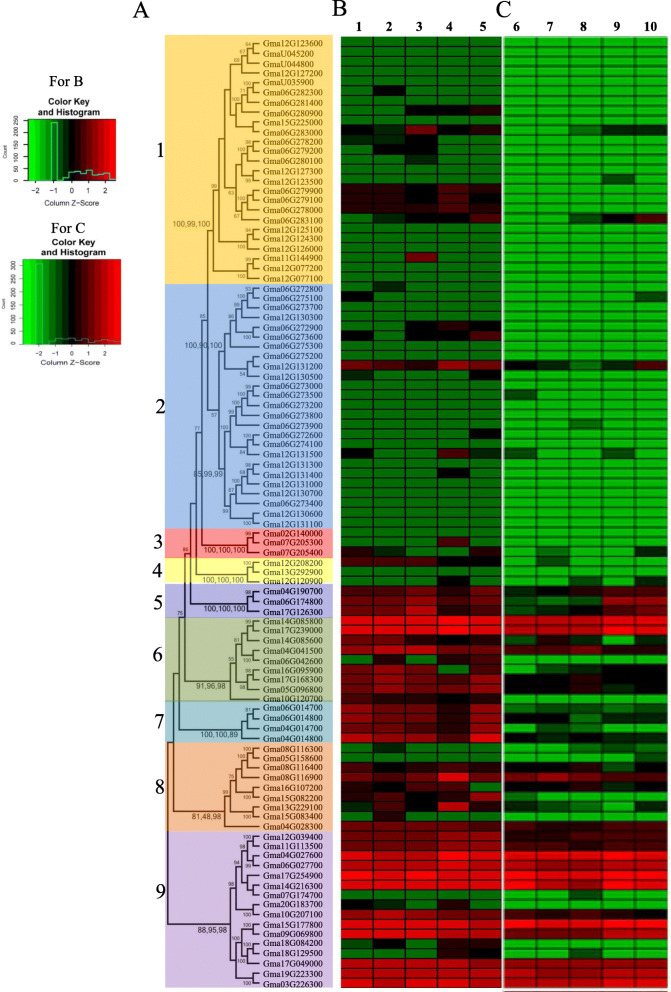
Phylogenetic and expression analysis of the identified 97 *GmPLCPs*. **a** Phylogenetic analysis of the identified 97 *GmPLCPs*. Phylogenetic tree construction of *GmPLCPs* is based on the full-length deduced amino acid sequences using the programs RAxML, MEGA version 6.0 and MrBayes 3.2. The tree shows nine major phylogenetic classes (class 1 to class 9) indicated with different shadows. **b** Expression analysis of the identified 97 *GmPLCPs* in five RNS-related tissues in the Phytozome database. The expression values of *GmPLCPs* in the Phytozome database and the pheatmap packages in R were used to product the Heatmap. These tissues include Nodules (1), Root Hairs (2), Roots (3), Nodules. Symbiotic. Condition (4) and Roots. Symbiotic. Condition (5). **c** RNA-seq analysis of the identified 97 *GmPLCPs* in five different nodules of soybean. Our previous RNA-Seq data [18] and the pheatmap packages in R were used to product this Heatmap. These five nodule samples include the nodules in branching stage (6), flowering stage (7), fruiting stage (8), pod stage (9) and harvest stage (10), and the description of these five important developmental stages were shown in our previous research [18]

To determine the phylogenetic relationships among the different members of soybean *PLCPs*, we performed a phylogenetic analysis based on the 97 full-length PLCP peptide sequence alignments. Combining with the classification in Fig. 2, these 97 soybean *PLCPs* were divided into 9 classes, for example, the soybean *PLCPs* in Group A were classified in class 1, the soybean *PLCP*s in Group B were classified in class 2, and so on (Fig. 6a).

To determine which soybean *PLCPs* were involved in RNS, we firstly investigated the expression profiles of the 97 soybean *PLCPs* in symbiosis-related tissues based on plant phytozome database, and these tissues included roots, root hairs, nodules, nodules. Symbiotic condition and root. Symiotic condition. The results showed that soybean *PLCPs* have distinct expression patterns in these tissues, and most of the highly expressed genes mainly focused on class 6 to class 10 (Fig. 6b). Then, we used our previous RNA-Seq data [18] to analyse the expression profiles of these 97 soybean *PLCPs* in five different nodules according to our previous RNA-Seq data (Fig. 6c). We compared the expression levels of these 97 soybean *PLCPs* in above-mentioned symbiosis-related tissues and nodule samples, and founded that among the highly expressed genes in symbiosis-related tissues, some *PLCPs* also had high expression in nodule samples (Fig. 6b and c), indicating that these *PLCPs* may participate in RNS or nodule development.

**Fig. 7:**
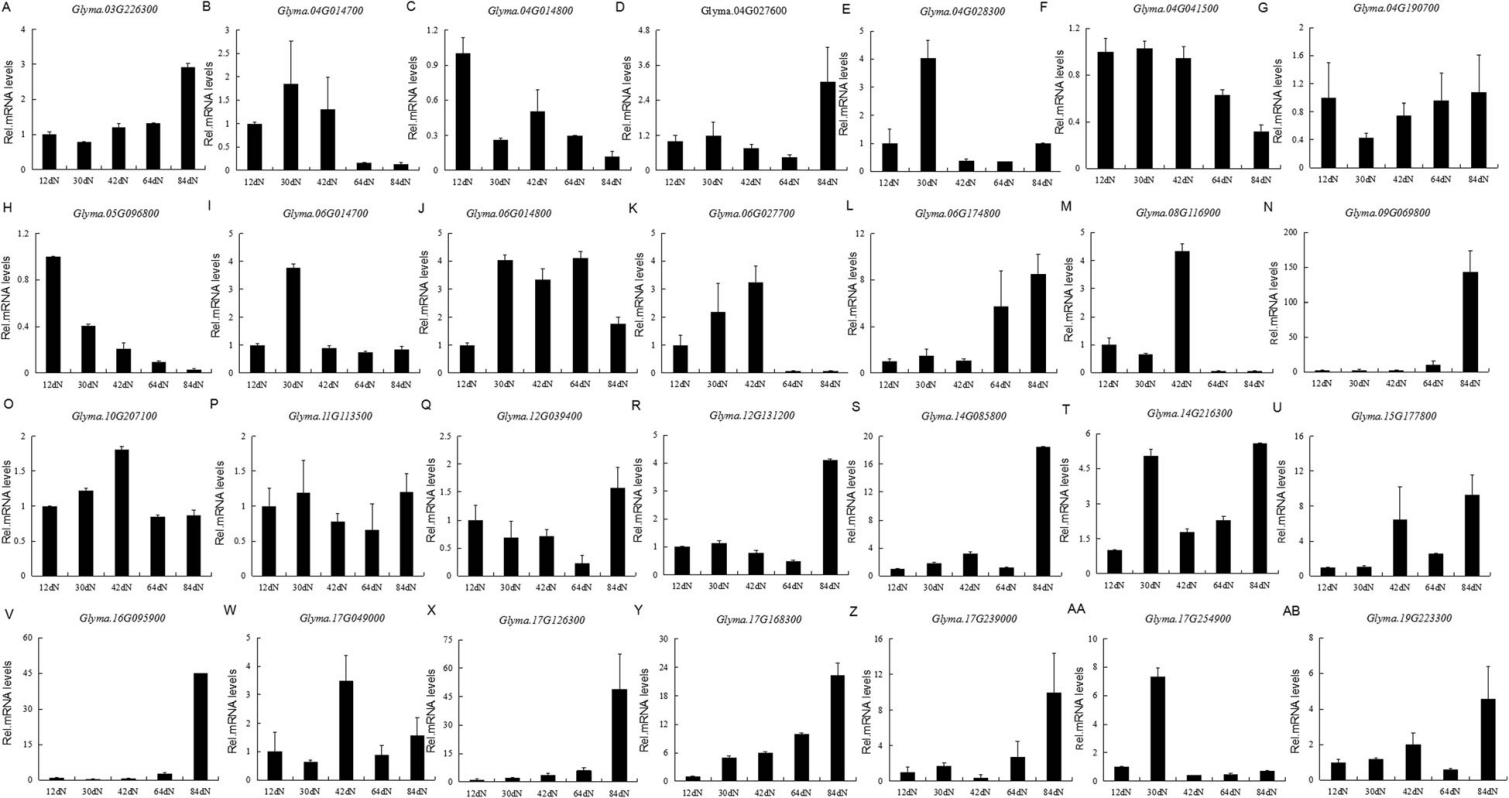
qPCR analysis of 28 selected *GmPLCPs* in nodule development. Soybean *ELF1b* and *QACT* were selected as reference genes to normalize the relative expression levels of each *GmPLCP* gene, and the expression level in 12dN was used as a starting point control to calculate the relative expression levels of other nodule samples. All qPCR reactions were repeated three times and the data are presented as the mean ± SD

To exam whether these *PLCPs* with high expression both in symbiosis-related tissues and nodule samples have roles in nodule development and/or senescence, we analyzed the expression difference of 28 selected *PLCPs* between different nodule development time points by qPCR. Firstly, the expression stability of four reference genes (*ELF1b*, *QACT*, *G6PD* and *Ubiquitin*) was evaluated, of which, *ELF1b* and *QACT* were most stable in all samples, while *GmG6PD* and *Ubiquitin* were consistently unstable (Fig. S1). Then, *ELF1b* and *QACT* were selected as reference genes for qPCR experiment and the results showed that nearly all of these 28 *PLCPs* were differentially expressed between the five nodules (Fig. 7). Among them, 12 *PLCPs* were up-regulated during nodule development and/or senescence, and reached their peaks at nodules collected at 84 days of post inoculation (84dN) (Fig. 7a, d, g, l, n, r, s, u, v, x, y and ab). Four *PLCPs* were down-regulated during nodule development, and had low expression at 64dN or 84dN (Fig. 7b, c, f and h). Seven *PLCPs* reached their peaks at 30dN or 42dN (Fig. 7e, I, k, m, o, w and aa). Three *PLCPs* were down-regulated then up-regulated during nodule development and/or senescence (Fig. 7p, q and z). *Glyma.06G014800* was up-regulated then down-regulated during nodule development and/or senescence (Fig.7j), and *Glyma.14G216300* had high expression at 30dN and 84dN (Fig. 7t).

### Functional analysis of *Glyma.04G190700* in soybean by hairy root transformation

**Fig. 8:**
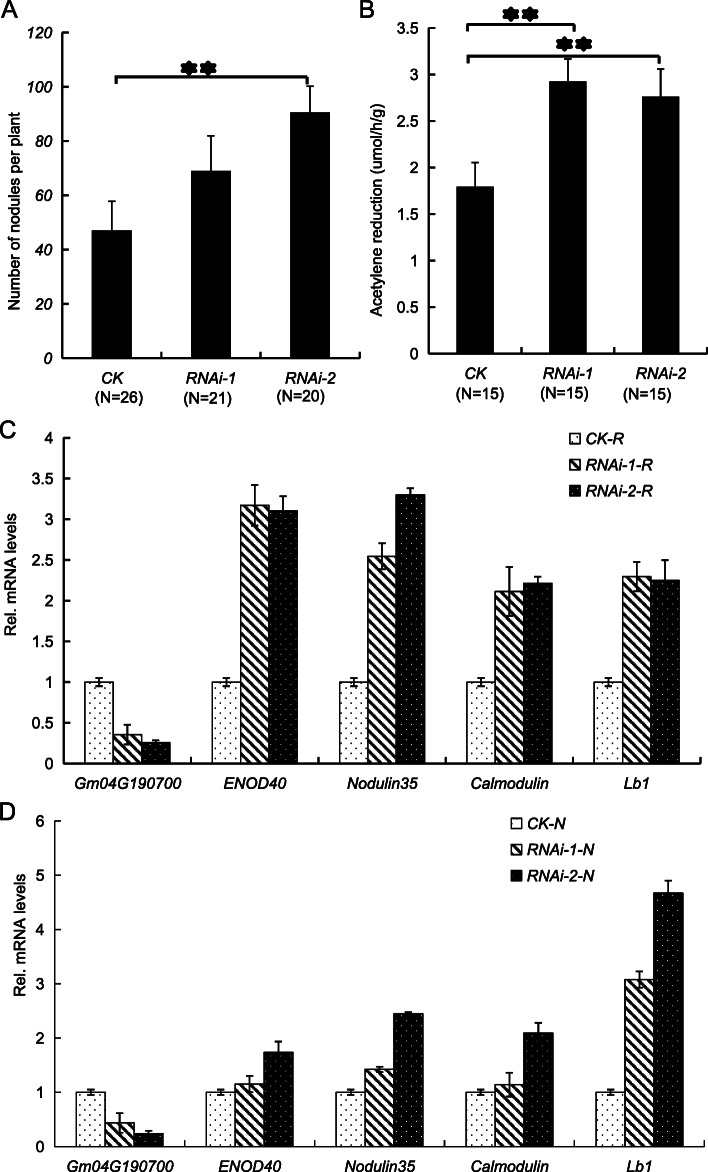
Effect of *Glyma.04G190700* RNAi on RNS in soybean. **a** Mean numbers of nodules per plant with altered *Glyma.04G190700* transcript levels. The numbers of independent transgenic plants in each sample are indicated in parentheses. **b** Nitrogenase activities of nodules in control and *Glyma.04G190700* RNAi plants. The vector p5941-35S-intron-(GFP-Bar marker) transgenic hairy roots were served as control (**a** and **b**). **c** qPCR analysis of the transcript levels of *Glyma.04G190700*, *ENOD40*, *Nodulin35*, *Calmodulin* and *Lb1* in the control and transgenic hairy roots. **d** qPCR analysis of the transcript levels of *Glyma.04G190700*, *ENOD40*, *Nodulin35*, *Calmodulin* and *Lb1* in the control and transgenic nodules. Total mixed RNA isolated from root system (including small nodules) or nodules of multiple independent transgenic plants was used for qPCR analysis. The references in the control hairy roots and nodules were used to calculate relative expression levels of these five genes in transgenic hairy roots and nodules. The data are presented as the mean ± SD and “  ” indicates statistical difference between different inoculated hair roots (t test, *p* < 0.01)

As described above, *Glyma.04G190700* was highly expressed in symbiosis-related tissues (Fig. 6b) and up-regulated during nodule development (Fig. 6c and Fig. 7g), suggesting that *Glyma.04G190700* may play a role in nodulation and nodule development. To confirm this result, RNA interference (RNAi) of *Glyma.04G190700* was performed using the soybean hairy root transformation method (Fig. 8). The symbiotic phenotypes were scored at 50 days after inoculation with BXYD3*.* RNAi of *Glyma.04G19070*0 resulted in increase in nodule number (Fig. 6a). Nodules in RNAi of *Glyma.04G190700* showed significantly higher nitrogenase activities than in the control (Fig. 6b). The expression levels of *Glyma.04G190700* and four nodulin genes (*ENOD40*, *Nodulin35*, *Calmodulin* and *Lb1*) [31–33] were examined by qPCR in hairy roots and nodules (Fig. 8c and d). The *Glyma.04G190700* transcript was reduced to less than 50% in the RNAi hairy roots and nodules as compared with that in the control (Fig. 8c and d). The expression levels of the four nodulin genes (*ENOD40*, *Nodulin35*, *Calmodulin* and *Lb1*) were increased drastically in the *Glyma.04G190700* RNAi hairy roots and nodules as compared with those in the control hairy roots and nodules (Fig. 8c and d). Together, these results strongly indicate that *Glyma.04G190700* participate in nodulation and nodule development.

## Discussion

*PLCPs* are a large class of proteolytic enzymes and play important roles in RNS [7, 8], while the whole-genome studies of *PLCP* family genes in legume are quite limited. In the present study, we firstly performed the whole-genome survey of soybean *PLCP* genes and explored the expansion of soybean *PLCP* family genes. The resultant expression patterns of *GmPLCPs* in soybean RNS provided insights into the putative roles of the legume *PLCPs* in nodulation, nodule development and senescence.

### Genome-wide scan and the expansion of soybean *PLCP* family genes

Genome-wide identify of *PLCPs* has been systematically performed in *Arabidopsis*, rubber, cassava, castor, poplar, grapevine, *G. hirsutum*, *C. papaya* and rice [1, 11–14]. In the present study, 97 *GmPLCPs* were identified in soybean, and the number is more than that of the other plants in the previous studies. According to the phylogenetic clade and structure features, these soybean *PLCPs* were classed into nine subfamilies, which were similar to previous studies [11, 34–36]. However, among these nine Groups, three Groups were formed with only legume *PLCPs* and only 26 *At PLCPs* in the Groups, which is greatly different from other studies [1, 11, 12].

Two whole genome duplication (WGD) events, occurring approximately 59 and 13 Ma, have been undergone in the soybean genome [37–39], and have significant contribution to the expansion of many multigene families in soybean [28, 40, 41]. Different gene families may have distinct polyploidy-derived duplicate events and the evolutionary mechanisms of the retention of duplicate genes [28], which play important roles in adaptive evolution and biological functions of families [42–44]. For soybean *PLCP* gene family, the number was significantly more than that in *A. thaliana*, *M. trunctula* and *L. japonicus*, which may result from WGD events, tandem duplication and large-scale segmental duplication [11, 12]. In the duplicate events of the soybean *PLCP* gene family two large-scale segmental duplication pairs included 37 *GmPLCPs* and greatly contributed to the family expansion, small tandem duplication pairs and single-gene segmental duplication pairs also have contribution in the expansion of soybean *PLCP* family genes. Besides, the retention of these repeat clusters genes might be mainly attributed to their different expression patterns, special structures and functions among these pairs, which was similar to the previous studies [45–47].

### Potential symbiotic function of *GmPLCPs* in soybean

Previous studies have shown that *PLCPs* play important roles in endogenous protein turnover [48], seed traits, germination and maturation [49, 50], abiotic environmental stresses [11, 49] and protection of plants against mites [51], fungi [52, 53], bacteria [54] and viruses [55]. The type III secretion system (T3SS) of rhizobia, an introducer of virulence factors from plant pathogens, can be induced by legume-derived flavonoid and has been reported to modulate nodulation process through recognition by the host defence system [56]. Besides, our previous research has shown that nodule development and senescence are directly associated with the plant immunity defence [26]. *PLCPs* have been reported to involve in biotic and abiotic stresses [11] and responsible for defense against pathogen bacteria and regulate plant immunity [54], and more and more molecular mechanisms were discovered in recent years [11]. In the present study, it was worth noting that no large tandem duplication pairs and only two small tandem duplication pairs of *GmPLCPs* were defferent expression in nodule development, suggesting that the RNS-related *GmPLCP* duplicate genes were mainly derived from single-gene segmental duplication rather than tandem duplication. Both homologous genes in the RNS-related duplicate pairs showed similar expression pattern in the RNS, which is different from the previous research [11].

Previous studies have been suggested that *PLCPs* play important roles in the development and senescence of several legume root nodules [17, 20–24, 57]. In soybean, *PLCPs* may play important roles in nodulation [15, 27] as well as nodule development and/or senescence [25, 26]. However, the roles of *GmPLCPs* in nodulation, nodule development and senescence are not fully understood. In the present study, the expression profiles of *GmPLCPs* in five symbiosis-related tissues and five different nodule samples [18] were comprehensively analyzed, and the results identified dozens RNS-related *PLCPs*, suggesting that multiple *GmPLCPs* may participate in nodulation signal recognition and immunity and/or nodule development and senescence. In the previous transcriptional profile data, the expressions of 14 *GmPLCPs* were particularly increased during the onset of senescence [25]. Among these *GmPLCPs*, 12 genes were up-regulated during nodule senescence, while the rest two genes had low expression at 64dN or 84dN. In this study, the other four *GmPLCPs* may also play roles in nodule development and senescence. Besides, five *PLCPs* may participate in the nitrogen-fixation process, and eight *PLCPs* may participate in the early and/or middle stage of nodule development. These data indicated that *GmPLCPs* may not only have roles in nodule senescence, but also participate in nodulation and nodule development. In this study, the symbiosis function analyses of *Glyma.04G190700* showed that it really participates in nodulation and nodule development. The specific regulatory role and distinct functions of RNS-related *GmPLCPs* need to be further studied.

### Characteristics of the RNS-related *GmPLCPs*

Previous studies have shown that polyploidy-derived duplicate genes lead to enhanced RNS in legumes [58, 59]. In the present study, RNS-related *GmPLCPs* were mainly focused on class 5 to class 9, and most of these genes participated in single-gene segmental duplication, indicating that polyploidy-derived duplicate event of *GmPLCPs* also played important roles in RNS. Besides, RNS-related *GmPLCPs* possessed relatively complex gene structures containing UTR sequences and three or more introns. Three divergent motif patterns were observed in these RNS-related GmPLCPs. The first pattern contained a propeptide_C1 domain [60] and a peptidase _C1A_cathepsin B domain [61] (only for Glyma.03G226300 and Glyma.19G223300). In the second pattern, which was found in three proteins (Glyma.04G028300, Glyma.14G085800 and Glyma.17G239000), had not only two typical motifs (an inhibitor _I29 domain and a peptidase _C1 domain) [62], but also a GRAN domain in their C-terminal regions, which was similar to some known PLCPs [10]. The rest or the most of these RNS-related GmPLCPs were generally categorized into the third pattern, which contained two typical motifs. These results indicated that there was no special protein structure in RNS-related GmPLCPs, which was similar to the nodulation and nodule development-related soybean cystatins [63].

## Conclusions

In summary, we conducted a genome-wide survey and identified 97 *GmPLCPs*. A total of 19 segmental duplication pairs created by WGD event were identified and analyzed, suggesting a high degree of complexity in the duplications of soybean *PLCPs*. Expression profiles of *GmPLCPs* in soybean root nodule symbiosis were used to identify the RNS-related *PLCPs*. The symbiosis function analyses showed that a RNS-related *GmPLCP* gene (*Glyma.04G190700*) really participate in nodulation and nodule development. Our findings improve our understanding of the functional diversity of legume *PLCP* family genes, and provide insights into the putative roles of the legume *PLCPs* in nodulation, nodule development and senescence.

## Methods

### Identification of *PLCPs* in soybean, *M. truncatula*, *L. japonicus* and *A. thaliana* and gene structure analysis

The *PLCP* family genes in soybean were identified from the Soybean Genome Database [http://soybase.org/] and the *Glycine max* Wm82.a2.v1 Phytozome Database [http://www.phytozome.net/soybean]. All of the identified GmPLCPs were then analyzed by NIH/NLM/NCBI CD-search tool, sequences without peptidase_C1 domain or the presence of large fragment deletion in the peptidase_C1 domain and/or the sequence length of < 150 amino acids were considered as putative pseudogenes and removed manually (Table S1 and Table S2). Basic Local Alignment Search Tool algorithms (BLASTP) with a threshold of e-value <1e-10 was used to identify the homologues of *GmPLCPs* in *M. truncatula*, *L. japonicus* and *A. thaliana*. The exon/intron/UTR structures of *PLCPs* were analyzed by using the gene structure display server program GSDS2.0 (http://gsds.cbi.pku.edu.cn/).

### Soybean *PLCPs* sequence

Phytozome v12.0 Database was searched to download the sequences of *GmPLCPs*. ExPasy site (http://web.expasy.org/protparam/) was used to calculate the isoelectric point (pI) and molecular weight of *GmPLCPs*. Map Chart software and the soybean genome annotation file (Gmax_275_Wm82.a2.v1.gene.gff3) were used to analysis the locations of *GmPLCPs* on chromosome. The SoyBase and the Soybean Breeder’s Toolbox (https://soybase.org/gb2/gbrowse/gmax2.0/) was used to obtain the blocks regarded as recent duplications.

### Phylogenetic analysis

The different *PLCPs* were applied for multi-species phylogenetic analysis including 97 *GmPLCPs*, 26 *PLCPs* from *A. thaliana*, 33 *PLCPs* from *M. trunctula*, and 25 from *L. japonicus*. Clustal W program was used to conduct the full-length peptide sequence alignments. MEGA6 software [64], Neighbor-Joining (NJ) method and 1000 bootstrap replicates analysis with the p-distance model were used to perform the multi-species phylogenetic tree. The programs RAxML, MEGA version 6.0 and MrBayes 3.2 (http://www.megasoftware.net) [64–66] were used to perform the phylogenetic of 97 *GmPLCPs*. RAxML 8.0.0 [67] was used to perform the Maximum likelihood (ML) analysis, the 1000 bootstrap replicates convergence test using the extended majority-rule consensus tree criterion (auto MRE) in RAxML was used to perform rapid 1000 bootstrap replicates analysis, and mixed model was used to construct the MrBayes analysis.

### Identification and analysis of duplicate segments pairs formed by soybean WGD events

To identify the duplicate segments pairs formed by soybean WGD events, firstly, the synonymous (Ks) of each *GmPLCP* or the duplicate gene pairs of *GmPLCPs* was identified from the SoyBase and the Soybean Breeder’s Toolbox. Secondly, according to the distribution of the *GmPLCPs* on the soybean chromosomes and their values of synonymous, eleven tandem repeat gene clusters were identified (Fig. 1). Thirdly, two big paralogous clusters (pairs No. 10 and 11) with two big tandem repeat gene clusters in each pair, five paralogous clusters (pairs No. 1, 9, 15, 17 and 19) with one tandem repeat gene cluster in each pair and 12 paralogous gene pairs were preliminary identified. Then to examine these preliminary identified 19 candidate paralogous segments pairs, the following two criterions were used in this study: 1) Duplicated segments pairs were grouped together in the *GmPLCPs* phylogenetic tree (Fig. 3 and Fig. 6a), and 2) the flanking regions of candidate duplicated segments pairs were showed in the collinearity analysis (Fig. 2).

The divergence times (T) were calculated using T = Ks/ (2 × 6.1 × 10^− 9^) × 10^− 6^ Mya [68] to estimate the date of the duplication pairs. Besides, the non-synonymous (Ka) of the paralog pairs was calculated using theYN00 method of the PAML program [69] to investigate the positive Darwinian selection in divergence following duplication. The ratio of Ka to Ks (ω = Ka/Ks) was calculated to measure the history of selection acting on coding sequences [28].

### Go annotation and gene expression analysis

The Go annotation of *GmPLCPs* was conducted by using the “Go Term Enrichment Tool” in Soybean Genome Database [http://soybase.org/]. The soybean database (https://soybase.org/ goslimgraphic_v2 /dashboard. php) was searched to download the detail gene information of these Go terms. The plant Phytozome database (Phytozome 12, http://www.phytozome.net/ soybean) was used to download the expression patterns data of *GmPLCPs* in five symbiosis-related tissues. The expression patterns of *GmPLCPs* in five nodule samples were analyzed by searching our previous RNA-seq data [26]. The pheatmap packages in R [41] were used to produce the heatmaps of these *GmPLCPs*.

### Plant materials and growth conditions

The surface-sterilized soybean Tianlong No.1 seedlings were germinated on moistened filter paper in a greenhouse, in which the day/night cycle was maintained at 16/8 h and the relative humidity (RH) at 70%, at 28 °C for 2–3 d. The seedlings were then grown in pots filled with sterilized perlite and vermiculite in proportion of 1:1, and watered with half-strength B&D medium [63]. After 4–5 d, soybean rhizobium 113–2 strain (stored in our lab) was used to inoculate the seedlings. Samples for RNA isolation were collected from soybean nodules at five time points: 12dN (nodules at 12 days after inoculation), 30dN (nodules at 30 days after inoculation), 42dN (nodules at 42 days after inoculation), 64dN (nodules at 64 days after inoculation) and 84dN (nodules at 84 days after inoculation). Nodules from different time points were separately collected with three biological replicates and were frozen at − 80 °C for RNA isolation.

### RNA extraction and qPCR

We used TRIzol reagent (Invitrogen, USA) to extract the total RNA of nodules, DNase I (Takara) to digest the total RNA, and a Prime Script RT reagent Kit (Perfect Real Time) with gDNA Eraser (Takara Bio, Inc) to perform the reverse-transcribed analysis. RNA quantity and quality were measured using an Epoch Multi-Volume Spectrophotometer system, NanoDrop and Agilent 2100 Bioanalyzer (Agilent Technologies, Palo Alto, CA, USA), and qPCR reactions on the five RNA samples were used to confirm the absence of gDNA in these RNA samples. cDNA from the reverse transcription of approximately 1 μg of RNA was used as the template for qPCR using primer sets listed in Table S4. RT-PCR amplification mixtures (20 μl) contained 2 μl template cDNA, iTaq Universial SYBR Green Supermix (10 μl) (Applied Biosystems) and 0.5 μl forward and reverse primer. Reactions were run on a CFX Connect Real-Time System (Applied Biosystems), and each assay included a no-template control (negative control). The cycling conditions of 30 s at 95 °C followed by 40 cycles of 5 s at 95 °C, 15 s at 60 °C and 12 s at 72 °C and final 5 s at 72 °C. After PCR amplification, a melting curve was generated for every PCR product to check the specificity of the PCR reaction (Fig. S2). The expression stability of four reference genes (*ELF1b*, *QACT*, *G6PD* and *Ubiquitin*) was evaluated, and *ELF1b* and *QACT* were selected as reference genes for the qPCR analyses of 28 selected *GmPLCPs*. Sample cycle threshold (CT) values were standardized for each template using the two reference gene as control, and the geNorm method [70] ; E, PCR efficiency) was used to analyze the relative changes in gene expression from the qPCR experiments. Three biological replica samples and three or more technical replicate reactions per sample were used to ensure statistical credibility.

### Glyma.04G190700-specific RNAi

A 206-bp fragment of the 5^′^-region of *Glyma.04G190700* was amplified by PCR and cloned into p5941-35S-intron-(GFP-Bar marker), generating p*Glyma.04G190700*-RNAi-1; A 185-bp fragment of the 3′-region of *Glyma.04G190700* was amplified by PCR and cloned into p5941-35S-intron-(GFP-Bar marker), generating p*Glyma.04G190700*-RNAi-2. In these two *Glyma.04G19070*0-Specific RNAi vectors, the sense and antisense *Glyma.04G190700* RNA sequences would be linked in tandem separated by the intron. The primers for the construction of these two *Glyma.04G190700*-Specific RNAi vectors were listed in Table S5.

*A. rhizogenes* cells K599 harboring p*Glyma.04G190700*-RNAi-1, p*Glyma.04G190700*-RNAi-2 and empty vector were used to induce formation of transgenic hairy roots in soybean. Transgenic hairy roots expressing the empty vector were used as a control. After inoculation with BXYD3, plants with positive transgenic hairy roots were grown for 50 days and nodulation phenotypes were scored. The expression level of *Glyma.04G190700*, *ENOD40*, *Nodulin35*, *Calmodulin* and *Lb1* in p*Glyma.04G190700*-RNAi or control was determined by qPCR using the primers listed in Table S5 and the procedure as described previously [71, 72]. Nitrogenase activity was determined by the acetylene reduction assay (ARA) as described by gas chromatography (GC-14, Japan) [73].

### Soybean hairy root transformation

*Glyma.04G190700*-specific RNAi constructs were transferred into *A. rhizogenes* K599 by electroporation, and then an *A. rhizogenes*-mediated procedure [74] was used to induce soybean hairy root formation. After infection, the soybean seedlings were transplanted to hydroponics containing soybean total nitrogen nutrition solution and covered with a transparent plastic to ensure high humidity. Removed the cotyledons after callus formation (about 5 days) and transplanted the seedlings to large hydroponic tanks. Within two weeks, hairy roots started to sprout from the site of infection and were screened with a fluorescence microscopy (Fig. S3). We then extracted the genomic DNA from the transgenic hairy roots to confirm CK and RNAi through gene specific primers. Each seedling was allowed to have only one transgenic hairy root and wrapped with ropes. For nodulation assays, transgenic composite plants were inoculated with *Rhizobium* BXYD3 and transferred to pots filled with vermiculite with 1/10 N (530 mmol/LN) and grown in a chamber in a 16−/8-h day/night cycle at 26 °C. We scored the nodulation phenotypes of these transgenic composite plants at 50 days after BXYD3 inoculation and used empty vector transgenic hairy roots as the control.

## Supplementary Information

**Additional file 1: Table S1.** The detailed information of soybean *PLCPs*.

**Additional file 2: Table S2.** The conserved domain analysis of soybean PLCPs by NIH/NLM/NCBI CD-search tool.

**Additional file 3: Table S3.** The gene ID information of the 42 GO terms in soybean *PLCPs*.

**Additional file 4: Table S4.** Primers for qPCR analysis of the 28 selected *GmPLCPs*.

**Additional file 5: Table S5.** Primers for *Glyma.04G190700*-specific RNAi analysis.

**Additional file 6: Fig. S1.** The stability assay of four references genes in five soybean nodule samples.

**Additional file 7: Fig. S2.** Melting curves of the primers of the 28 selected *GmPLCPs*.

**Additional file 8: Fig. S3.** Screening of the positive transgenic hairy roots.

## Data Availability

All data and material used in this study are available from the corresponding author upon reasonable request.

## Abbreviations

PLCP: Plant papain-like cysteine protease
RNS: Root nodule symbiosis
WGD: Whole-genome duplication
GRAN: Granulin
pI: Isoelectric point
Ks: Synonymous mutation rate
Ka: Non-synonymous mutation rate
RNAi: RNA interference
NJ: Neighbor-Joining
ML: Maximum likelihood
RH: Relative humidity
BLASTP: Basic Local Alignment Search Tool algorithms
T3SS: Type III secretion system

## References

[1] Liu H, Hu M, Wang Q, Cheng L, Zhang Z (2018). Role of papain-like cysteine proteases in plant development. Front Plant Sci.

[2] Shindo T, Van der Hoorn RA (2008). Papain-like cysteine proteases: key players at molecular battlefields employed by both plants and their invaders. Mol Plant Pathol.

[3] Misas-Villamil JC, van der Hoorn RA, Doehlemann G (2016). Papain-like cysteine proteases as hubs in plant immunity. New Phytol.

[4] Díaz-Mendoza M, Velasco-Arroyo B, González-Melendi P, Martínez M, Díaz I (2014). C1A cysteine protease-cystatin interactions in leaf senescence. J Exp Bot.

[5] Grudkowska M, Zagdańska B (2004). Multifunctional role of plant cysteine proteinases. Acta biochim po.

[6] Rabbani MA, Maruyama K, Abe H, Khan MA, Katsura K, Ito Y (2003). Monitoring expression profiles of rice genes under cold, drought, and high-salinity stresses and abscisic acid application using cDNA microarray and RNA gel-blot analyses. Plant Physiol.

[7] Kempema LA, Cui X, Holzer FM, Walling LL (2007). Arabidopsis transcriptome changes in response to phloem-feeding silverleaf whitefly nymphs. Similarities and distinctions in responses to aphids. Plant Physiol.

[8] Rehm FBH, Jackson MA, De Geyter E, Yap K, Gilding EK, Durek T (2019). Papain-like cysteine proteases prepare plant cyclic peptide precursors for cyclization. PNAS..

[9] Rawlings ND (2010). Peptidase inhibitors in the MEROPS database. Biochimie..

[10] Bateman A, Bennett HP (2009). The granulin gene family: from cancer to dementia. BioEssays.

[11] Zhang S, Xu Z, Sun H, Sun L, Shaban M, Yang X (2019). Genome-wide identification of papain-like cysteine proteases in gossypium hirsutum and functional characterization in response to verticillium dahliae. Front Plant Sci.

[12] Liu J, Sharma A, Niewiara MJ, Singh R, Ming R, Yu Q (2018). Papain-like cysteine proteases in Carica papaya: lineage-specific gene duplication and expansion. BMC Genomics.

[13] Wang W, Zhou XM, Xiong HX, Mao WY, Zhao P, Sun MX (2018). Papain-like and legumain-like proteases in rice: genome-wide identification, comprehensive gene feature characterization and expression analysis. BMC Plant Biol.

[14] Beers EP, Jones AM, Dickerman AW (2004). The S8 serine, C1A cysteine and A1 aspartic protease families in *Arabidopsis*. Phytochemistry..

[15] Quain MD, Makgopa ME, Cooper JW, Kunert KJ, Foyer CH (2015). Ectopic phytocystatin expression increases nodule numbers and influences the responses of soybean (*Glycine max*) to nitrogen deficiency. Phytochemistry..

[16] Sheokand S, Brewin NJ (2003). Cysteine proteases in nodulation and nitrogen fixation. Indian J Exp Biol.

[17] Mergaert P, Kereszt A, Kondorosi E (2020). Gene expression in nitrogen-fixing symbiotic nodule cells in *Medicago truncatula* and other nodulating plants. Plant Cell.

[18] Vincent JL, Brewin NJ (2000). Immunolocalization of a cysteine protease in vacuoles, vesicles, and symbiosomes of pea nodule cells. Plant Physiol.

[19] Kardailsky IV, Brewin NJ (1996). Expression of cysteine protease genes in pea nodule development and senescence. Mol Plant-microbe Interactions.

[20] Pierre O, Hopkins J, Combier M, Baldacci F, Engler G, Brouquisse R (2014). Involvement of papain and legumain proteinase in the senescence process of *Medicago truncatula* nodules. New Phytol.

[21] Deng J, Zhu F, Liu J, Zhao Y, Wen J, Wang T (2019). Transcription factor bHLH2 represses CYSTEINE PROTEASE77 to negatively regulate nodule senescence. Plant Physiol.

[22] Naito Y, Fujie M, Usami S, Murooka Y, Yamada T (2000). The involvement of a cysteine proteinase in the nodule development in Chinese milk vetch infected with *Mesorhizobium huakuii subsp. rengei*. Plant Physiol.

[23] Li Y, Zhou L, Li Y, Chen D, Tan X, Lei L (2008). A nodule-specific plant cysteine proteinase, *AsNODF32*, is involved in nodule senescence and nitrogen fixation activity of the green manure legume Astragalus sinicus. New Phytol.

[24] Alesandrini F, Mathis R, Sype GV, Hérouart D, Puppo A. Possible roles for a cysteine protease and hydrogen peroxide in soybean nodule development and senescence. New Phytol. 2003;158(1):131–8.

[25] van Wyk SG, Du Plessis M, Cullis CA, Kunert KJ, Vorster BJ (2014). Cysteine protease and cystatin expression and activity during soybean nodule development and senescence. BMC Plant Biol.

[26] Yuan SL, Li R, Chen HF, Zhang CJ, Chen LM, Hao QN (2017). RNA-Seq analysis of nodule development at five different developmental stages of soybean (*Glycine max*) inoculated with *Bradyrhizobium japonicum* strain 113-2. Sci Rep.

[27] Yuan S, Li R, Chen S, Chen H, Zhang C, Chen L (2016). RNA-Seq analysis of differential gene expression responding to different rhizobium strains in soybean (*Glycine max*) roots. Front Plant Sci.

[28] Li S, Wang N, Ji D, Xue Z, Yu Y, Jiang Y (2016). Evolutionary and functional analysis of membrane-bound NAC transcription factor genes in soybean. Plant Physiol.

[29] Juretic N, Hoen DR, Huynh ML, Harrison PM, Bureau TE (2005). The evolutionary fate of MULE-mediated duplications of host gene fragments in rice. Genome Res.

[30] Freeling M (2008). The evolutionary position of subfunctionalization, downgraded. Genome dynamics.

[31] Minami E, Kouchi H, Cohn JR, Ogawa T, Stacey G (1996). Expression of the early nodulin, ENOD40, in soybean roots in response to various lipo-chitin signal molecules. Plant J.

[32] Legocki RP, Verma DP (1979). A nodule-specific plant protein (nodulin-35) from soybean. Science..

[33] Choudhury SR, Pandey S (2015). Phosphorylation-dependent regulation of G-protein cycle during nodule formation in soybean. Plant Cell.

[34] Richau KH, Kaschani F, Verdoes M, Pansuriya TC, Niessen S, Stüber K (2012). Subclassification and biochemical analysis of plant papain-like cysteine proteases displays subfamily-specific characteristics. Plant Physiol.

[35] Martinez M, Diaz I (2008). The origin and evolution of plant cystatins and their target cysteine proteinases indicate a complex functional relationship. BMC Evol Biol.

[36] Zou Z, Xie G, Yang L (2017). Papain-like cysteine protease encoding genes in rubber (*Hevea brasiliensis*): comparative genomics, phylogenetic, and transcriptional profiling analysis. Planta..

[37] Schlueter JA, Dixon P, Granger C, Grant D, Clark L, Doyle JJ (2004). Mining EST databases to resolve evolutionary events in major crop species. Genome..

[38] Schmutz J, Cannon SB, Schlueter J, Ma J, Mitros T, Nelson W (2010). Genome sequence of the palaeopolyploid soybean. Nature..

[39] Vanneste K, Baele G, Maere S, Van de Peer Y (2014). Analysis of 41 plant genomes supports a wave of successful genome duplications in association with the cretaceous-Paleogene boundary. Genome Res.

[40] Liu HJ, Tang ZX, Han XM, Yang ZL, Zhang FM, Yang HL (2015). Divergence in enzymatic activities in the soybean GST supergene family provides new insight into the evolutionary dynamics of whole-genome duplicates. Mol Biol Evol.

[41] Han Y, Li X, Cheng L, Liu Y, Wang H, Ke D (2016). Genome-wide analysis of soybean JmjC domain-containing proteins suggests evolutionary conservation following whole-genome duplication. Front Plant Sci.

[42] Adams KL, Wendel JF (2005). Polyploidy and genome evolution in plants. Curr Opin Plant Biol.

[43] De Smet R, Van de Peer Y (2012). Redundancy and rewiring of genetic networks following genome-wide duplication events. Curr Opin Plant Biol.

[44] Moghe GD, Shiu SH (2014). The causes and molecular consequences of polyploidy in flowering plants. Ann N Y Acad Sci.

[45] Ganko EW, Meyers BC, Vision TJ (2007). Divergence in expression between duplicated genes in Arabidopsis. Mol Biol Evol.

[46] Lan T, Yang ZL, Yang X, Liu YJ, Wang XR, Zeng QY (2009). Extensive functional diversification of the Populus glutathione S-transferase supergene family. Plant Cell.

[47] Yang ZL, Liu HJ, Wang XR, Zeng QY (2013). Molecular evolution and expression divergence of the Populus polygalacturonase supergene family shed light on the evolution of increasingly complex organs in plants. New Phytol.

[48] Martinez M, Cambra I, Carrillo L, Diaz-Mendoza M, Diaz I (2009). Characterization of the entire cystatin gene family in barley and their target cathepsin L-like cysteine-proteases, partners in the hordein mobilization during seed germination. Plant Physiol.

[49] Quain MD, Makgopa ME, Marquez-Garcia B, Comadira G, Fernandez-Garcia N, Olmos E (2014). Ectopic phytocystatin expression leads to enhanced drought stress tolerance in soybean (*Glycine max*) and *Arabidopsis thaliana* through effects on strigolactone pathways and can also result in improved seed traits. Plant Biotechnol J.

[50] Arai S, Matsumoto I, Emori Y, Abe K (2002). Plant seed cystatins and their target enzymes of endogenous and exogenous origin. J agr food chem.

[51] Carrillo L, Martinez M, Ramessar K, Cambra I, Castanera P, Ortego F (2011). Expression of a barley cystatin gene in maize enhances resistance against phytophagous mites by altering their cysteine-proteases. Plant Cell Rep.

[52] Martinez M, Lopez-Solanilla E, Rodriguez-Palenzuela P, Carbonero P, Diaz I (2003). Inhibition of plant-pathogenic fungi by the barley cystatin Hv-CPI (gene icy) is not associated with its cysteine-proteinase inhibitory properties. Mol Plant-microbe Interactions.

[53] Popovic M, Andjelkovic U, Burazer L, Lindner B, Petersen A, Gavrovic-Jankulovic M (2013). Biochemical and immunological characterization of a recombinantly-produced antifungal cysteine proteinase inhibitor from green kiwifruit (*Actinidia deliciosa*). Phytochemistry..

[54] Shindo T, Kaschani F, Fan Y, Kovács J, Fang T, Kourelis J (2016). Screen of non-annotated small secreted proteins of Pseudomonas syringae reveals a virulence factor that inhibits tomato immune proteases. PLoS Pathog.

[55] Gutierrez-Campos R, Torres-Acosta JA, Saucedo-Arias LJ, Gomez-Lim MA (1999). The use of cysteine proteinase inhibitors to engineer resistance against potyviruses in transgenic tobacco plants. Nature biotechnol.

[56] Okazaki S, Kaneko T, Sato S, Saeki K (2013). Hijacking of leguminous nodulation signaling by the rhizobial type III secretion system. PNAS..

[57] Perez Guerra JC, Coussens G, De Keyser A, De Rycke R, De Bodt S, Van De Velde W (2010). Comparison of developmental and stress-induced nodule senescence in *Medicago truncatula*. Plant Physiol.

[58] Young ND, Debellé F, Oldroyd GE, Geurts R, Cannon SB, Udvardi MK (2011). The Medicago genome provides insight into the evolution of rhizobial symbioses. Nature..

[59] Li QG, Zhang L, Li C, Dunwell JM, Zhang YM (2013). Comparative genomics suggests that an ancestral polyploidy event leads to enhanced root nodule symbiosis in the *Papilionoideae*. Mol Biol Evol.

[60] Chan SJ, San Segundo B, McCormick MB, Steiner DF (1986). Nucleotide and predicted amino acid sequences of cloned human and mouse preprocathepsin B cDNAs. PNAS..

[61] Turk D, Guncar G (2003). Lysosomal cysteine proteases (cathepsins): promising drug targets. Acta crystallographica section D. Biological crystallography.

[62] Liu LN, Cui J, Zhang X, Wei T, Jiang P, Wang ZQ (2013). Analysis of structures, functions, and epitopes of cysteine protease from *Spirometra erinaceieuropaei Spargana*. Biomed Res Int.

[63] Yuan S, Li R, Wang L, Chen H, Zhang C, Chen L (2016). Search for nodulation and nodule development-related cystatin genes in the genome of soybean (*Glycine max*). Front Plant Sci.

[64] Tamura K, Stecher G, Peterson D, Filipski A, Kumar S (2013). MEGA6: molecular evolutionary genetics analysis version 6.0. Mol Biol Evol.

[65] Guindon S, Gascuel O (2003). A simple, fast, and accurate algorithm to estimate large phylogenies by maximum likelihood. Syst Biol.

[66] Ronquist F, Teslenko M, van der Mark P, Ayres DL, Darling A, Hohna S (2012). MrBayes 3.2: efficient Bayesian phylogenetic inference and model choice across a large model space. Syst Biol.

[67] Stamatakis A (2014). RAxML version 8: a tool for phylogenetic analysis and post-analysis of large phylogenies. Bioinformatics (Oxford, England).

[68] Lynch M, Conery JS (2000). The evolutionary fate and consequences of duplicate genes. Science (New York, NY).

[69] Yang Z (2007). PAML 4: phylogenetic analysis by maximum likelihood. Mol Biol Evol.

[70] Hellemans J, Mortier G, De Paepe A, Speleman F, Vandesompele J (2007). qBase relative quantification framework and software for management and automated analysis of real-time quantitative PCR data. Genome Biol.

[71] Yuan S, Zhu H, Gou H, Fu W, Liu L, Chen T (2012). A ubiquitin ligase of symbiosis receptor kinase involved in nodule organogenesis. Plant Physiol.

[72] Feng Y, Wu P, Fu W, Peng L, Zhu H, Cao Y (2020). The *Lotus japonicus* ubiquitin ligase SIE3 interacts with the transcription factor SIP1 and forms a homodimer. Front Plant Sci.

[73] Chen L, Qin L, Zhou L, Li X, Chen Z, Sun L (2019). A nodule-localized phosphate transporter *GmPT7* plays an important role in enhancing symbiotic N(2) fixation and yield in soybean. New Phytol.

[74] Kereszt A, Li D, Indrasumunar A, Nguyen CD, Nontachaiyapoom S, Kinkema M (2007). *Agrobacterium rhizogenes*-mediated transformation of soybean to study root biology. Nat Protoc.

