# Supplementary material for: Genome-wide survey of soybean papain-like cysteine proteases and their expression analysis in root nodule symbiosis
Source: BMC Plant Biol. 2020 Nov 12;20:517. doi: 10.1186/s12870-020-02725-5 (PMC7659060; doi:10.1186/s12870-020-02725-5)
Supplement: Supplementary file 6 — Additional file 6: Fig. S1. The stability assay of four references genes in five soybean nodule samples. [file 12870_2020_2725_MOESM6_ESM.pdf]

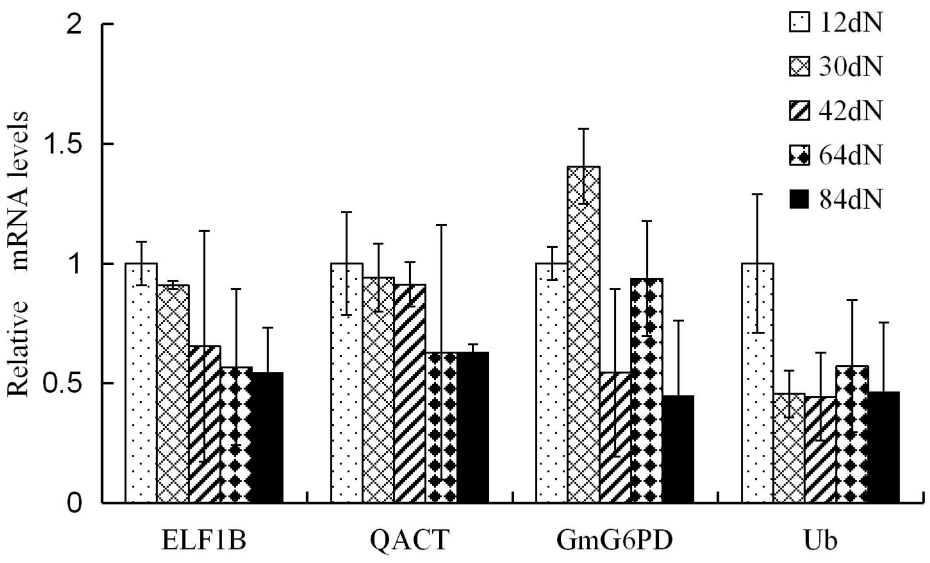

Supplemental Fig. S1 The stability assay of four references genes in five soybean nodule samples.
