# Supplementary figures and images for: Genome-wide survey of soybean papain-like cysteine proteases and their expression analysis in root nodule symbiosis
Source: BMC Plant Biol. 2020 Nov 12;20:517. doi: 10.1186/s12870-020-02725-5 (PMC7659060; doi:10.1186/s12870-020-02725-5)

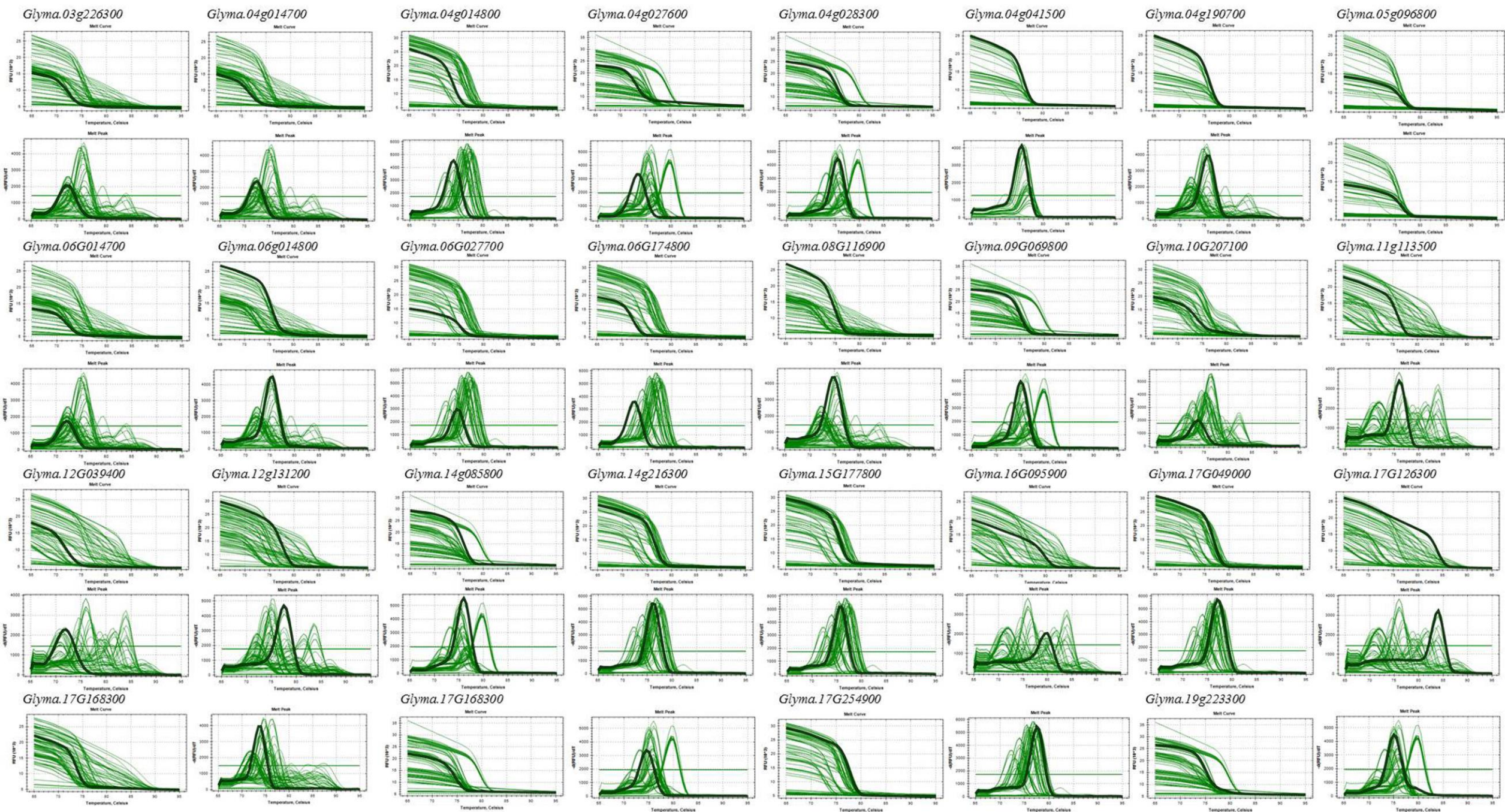

**Supplemental Fig. S2** Melting curves of the primers of the 28 selected *GmPLCPs*.

Supplement: Supplementary file 7 — Additional file 7: Fig. S2. Melting curves of the primers of the 28 selected GmPLCPs. [file 12870_2020_2725_MOESM7_ESM.pdf]
