# Supplementary material for: Genome-wide survey of soybean papain-like cysteine proteases and their expression analysis in root nodule symbiosis
Source: BMC Plant Biol. 2020 Nov 12;20:517. doi: 10.1186/s12870-020-02725-5 (PMC7659060; doi:10.1186/s12870-020-02725-5)
Supplement: Supplementary file 8 — Additional file 8: Fig. S3. Screening of the positive transgenic hairy roots. [file 12870_2020_2725_MOESM8_ESM.pdf]

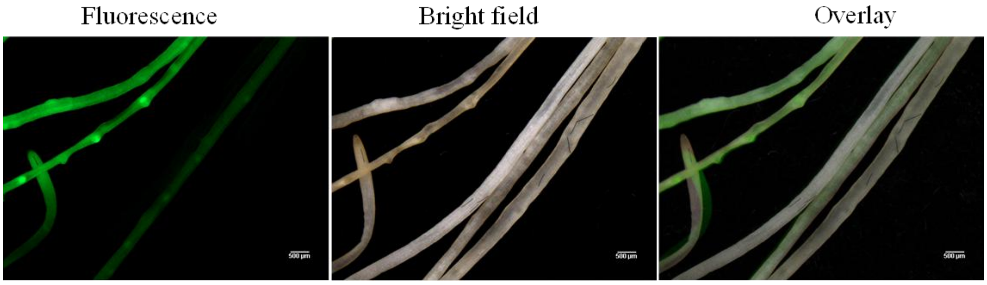

Supplemental Fig. S3 Screening of the positive transgenic hairy roots. Overlay images (right panel) were generated by superimpose of the fluorescence signal (left panel) with the bright-field images (middle panel).
